# Microstructure, Mechanical Properties and Corrosion Performance of Laser-Welded NiTi Shape Memory Alloy in Simulated Body Fluid

**DOI:** 10.3390/ma17194801

**Published:** 2024-09-29

**Authors:** A. Rajesh Kannan, N. Siva Shanmugam, V. Rajkumar, M. Vishnukumar, S. G. Channabasavanna, Junho Oh, Than Trong Khanh Dat, Jonghun Yoon

**Affiliations:** 1 Department of Mechanical Engineering, BK21 FOUR ERICA-ACE Center, Hanyang University, 55, Hanyangdaehak-ro, Sangnok-gu, Ansan 15588, Gyeonggi-do, Republic of Korea; rajeshkannan@hanyang.ac.kr (A.R.K.); wj6478@gmail.com (J.O.); 2 Department of Mechanical Engineering, National Institute of Technology, Tiruchirappalli 620015, Tamil Nadu, India; nsiva@nitt.edu; 3 Department of Mechanical Engineering, Coimbatore Institute of Engineering and Technology, Coimbatore 641109, Tamil Nadu, India; rajkmech42@gmail.com; 4 Department of Metallurgical and Materials Engineering, National Institute of Technology, Tiruchirappalli 620015, Tamil Nadu, India; carevishnu@gmail.com; 5 Department of Mechanical Engineering, Sri Jayachamarajendra College of Engineering, JSS Science and Technology University, Mysuru 570006, Karnataka, India; channasg1994@gmail.com; 6 Faculty of Mechanical Engineering, Ho Chi Minh City University of Technology (HCMUT), 268 Ly Thuong Kiet Street, District 10, Ho Chi Minh City 700000, Vietnam; ttkdat@hcmut.edu.vn; 7 Vietnam National University Ho Chi Minh City, Linh Trung Ward, Thu Duc City, Ho Chi Minh City 700000, Vietnam; 8 AIDICOME Inc., 55, Hanyangdaehak-ro, Sangnok-gu, Ansan 15588, Gyeonggi-do, Republic of Korea

**Keywords:** shape memory alloy, NiTi, laser-welding, microstructure, corrosion, simulated body fluid

## Abstract

Laser-welding is a promising technique for welding NiTi shape memory alloys with acceptable tensile strength and comparable corrosion performance for biomedical applications. The microstructural characteristics and localized corrosion behavior of NiTi alloys in a simulated body fluid (SBF) environment are evaluated. A microstructural examination indicated the presence of fine and equiaxed grains with a B2 austenite phase in the base metal (BM), while the weld metal (WM) had a coarse dendritic microstructure with intermetallic precipitates including Ti_2_Ni and Ni_4_Ti_3_. The hardness decreased from the BM to the WM, and the average hardness for the BM was 352 ± 5 HV, while it ranged between 275 and 307 HV and 265 and 287 HV for the HAZ and WM, respectively. Uni-axial tensile tests revealed a substantial decrease in the tensile strength of NiTi WM (481 ± 19 MPa), with a reduced joint efficiency of 34%. The localized corrosion performance of NiTi BM was superior to the WM, with electrochemical test responses indicating a pitting potential and low corrosion rate in SBF environments. The corrosion rate of the NiTi BM and WM was 0.048 ± 0.0018 mils per year (mpy) and 0.41 ± 0.019 mpy, respectively. During welding, NiTi’s strength and biocompatibility properties changed due to the alteration in microstructure and formation of intermetallic phases as a result of Ti enrichment. The performance and safety of welded medical devices may be impacted during welding, and it is essential to preserve the biocompatibility of NiTi components for biomedical applications.

## 1. Introduction

Nickel–Titanium (NiTi or Nitinol) shape memory alloys have been employed in numerous engineering sectors during the past few decades due to their superior shape memory effect, biocompatibility, and super elasticity characteristics [1,2]. NiTi alloys have been widely employed in biomedical, hydrospace, automotive, aerospace, and medical industries [3]. One of the most widely used industries for NiTi is biomedical engineering, particularly in fabricating orthodontic arc wires for cardiovascular stents, surgical tools, miniature devices, or orthopedic implants [4]. With regard to the mechanical integrity of the welded joints, fusion-based joining techniques such as gas tungsten arc welding [5] and laser-welding [6] have revealed the best outcomes. Laser-welding is the most-used welding technique over other fusion-based welding processes due to its characteristics, such as a reduced heat-affected region, deep and narrow fusion zone, better production rate, lower residual stress, higher energy density, and excellent control of process variables [7,8]. Laser-welding is a highly useful technique in aerospace [9,10], transportation [11,12], and biological [13,14,15] contexts because it can generate high-quality welds with few errors and is adaptable to many materials and complex geometries. During the welding, NiTi alloys may undergo a shift in the chemical composition, resulting in the transformation temperatures of thermally altered areas; i.e., after welding, the microstructure of the parent NiTi alloy with a fully austenitic structure may exhibit martensite or a combination of martensite and austenite in areas exposed to welding. These microstructural changes can significantly affect the mechanical integrity and corrosion performance of welded NiTi structures. Due to the poor formability and machinability characteristics, NiTi alloys are often limited in manufacturing small and miniature devices. Many studies have evaluated the mechanical integrity and corrosion performance of wrought NiTi alloys in different corrosive environments, while few studies have reported on the welded NiTi structures.

Oliveira et al. [1] studied the influence of laser-welding process variables on the fraction of austenite and martensite phases in NiTi alloys. The fraction of martensite varied from the base metal (BM) to the weld metal (WM) due to the higher thermal gradients during welding. Mehrpouya et al. [6] investigated the microstructural features, shape memory effect characteristics, and hardness of NiTi sheets via a diode laser. Epitaxial growth was noticed in the fusion zone, and the formation of precipitates such as NiTi_2_, Ni_3_Ti, and Ni_4_Ti_3_ was confirmed. Also, the microhardness of the welded NiTi alloy at the weld center (270–300 HV) showed a decrease in hardness compared to the BM (320 HV). Datta et al. [16] welded 1 mm NiTi sheets with a Yb-doped fiber laser system and explored the correlation between input–output responses using different artificial neural network (ANN) models. The output responses, such as the weld bead characteristics, hardness, and tensile properties, were evaluated. In addition, the microstructure varied from the BM to the WM, and a drastic drop in the tensile properties was observed, while the Bonobo optimizer showed less deviation in predicted values than that of other ANN models. Kannan et al. [17] evaluated the corrosion behavior of laser-welded NiTi sheets in a 0.9% NaCl solution at 37.5 °C. The corrosion behavior of the NiTi WM was better than that of the BM due to an increase in the Ti/Ni ratio. Prabu et al. [18] explored the functional and corrosion properties of friction-stir-welded NiTi sheets having 1.2 mm thickness. The average tensile strength and elongation of the NiTi WM was 605 MPa and 7% in comparison to the BM (990 MPa and 27%). Also, the localized corrosion behavior of the NiTi WM was reduced due to the microstructural difference across the weld, resulting in the formation of nonuniform oxide layers.

Biocompatibility of the NiTi alloys has been assessed under different simulated environments [19,20]. The release of Ni ions is the dominant factor that controls the compatibility and safe use of NiTi alloys in the human body. Concerning the present study, Sevilla et al. [21] examined the phase transformation, mechanical integrity, and corrosion characteristics of NiTi orthodontic archwires using Nd: YAG laser-welding for selective force application. After welding, the microstructure of NiTi did not change considerably. At the same time, tensile properties were reduced significantly, and the release of Ni ions in the artificial saliva medium was within the biological tolerance range. Toker et al. [4] studied the microstructural characteristics and localized corrosion performance of wrought NiTi alloys in a simulated body fluid (SBF) environment. The corrosion results indicated the formation of stoichiometric TiO_2_ oxides near high-energy zones like dislocation networks. Also, Ni- and Ti-rich intermetallic phases existed in the corroded surfaces and were attributed to the higher inner diffusion, followed by a Ni release. Kassab et al. [22] assessed the fracture of NiTi wires due to corrosion in simulated oral environments. The NiTi wires demonstrated sufficient resistance to corrosion in conditions with 9 g/L NaCl and SBFs, like saliva. In addition, the fracture type was brittle, while the corrosion pits acted as crack nucleation points. The corrosion resistance of NiTi alloys mainly depends on the passive film formation on the surface. However, the release of ions can significantly influence the life of bioimplants. Furthermore, the passive film may deteriorate throughout its service life. The physiological environment can influence the NiTi alloy’s corrosion performance and oxide products. The welding of NiTi alloys can alter the microstructure and intermetallic formation, which can influence the release of metal ions into the body, causing health issues. Beyond that, the existence of martensite influences the dislocation formation at the austenite and martensite phase boundaries, resulting in enhanced selective corrosion attacks. Most studies have reported optimizing process variables, microstructures, and mechanical properties of welded NiTi alloys. In this study, a novel attempt is made to evaluate the microstructural features, mechanical properties, and corrosion performance of laser-welded NiTi sheets. Electrochemical tests were performed in SBF, and a comparison was made between un-welded and laser-welded NiTi specimens. The interaction between the laser-welding process and the resultant alloy behavior is uniquely explored in this study, offering new insights into the material’s appropriateness for biomedical applications, especially concerning long-term implant durability and performance.

## 2. Materials and Methods

Annealed and oxide-free sheets of NiTi with a 1 mm thickness were used in the present study. The nominal composition of the NiTi sheets conforms to the elemental limits in accordance with ASTM F2063-18 standards [23], as presented in Table 1. Before welding, the coupons of NiTi were mechanically scrubbed with a stainless steel wire brush and then cleaned with acetone to remove the contaminants from the specimen surface. The power source for the welding process was a Yb-YAG fiber laser-welding system (Make: TRUMPF GmbH, Ditzingen, Germany) with 4 kW power, a 0.2 mm focal spot size, and a 1.03 μm wavelength. From iterative trial and error runs, the optimized set of process variables was selected to fabricate the coupons to a butt joint configuration. The coupons for welding were rigidly clamped without any gap. From existing literature, the criteria for selecting the processing variables to fabricate the joints were based on the weld bead characteristics, i.e., the weld bead with full penetration depth. The range for conducting trials was considered from an existing study [24], and the process variables were slightly modified to obtain better results in the present study. The process variables, such as the beam power (W) and welding speed (mm/min), were controlled in the range of 850 to 950 W and 2000 to 2500 mm/min, respectively. The optimized set of process variables is presented in Table 2. The top and bottom sides of the blanks to be welded were supplied with 99.99% pure industrial-grade argon at a constant flow rate of 15 L/min to prevent contamination in welds.

After welding, the weld bead region with a uniform cross-section was considered for specimen preparation using an electric discharge machining (EDM) process. The microstructural features and elemental distribution in NiTi’s BM and WM specimens were examined with a Zeiss (Zeiss, Oberkochen, Germany) field-emission scanning electron microscope (SEM) equipped with energy-dispersive X-ray spectroscopy (EDS). For microstructural analysis, the transverse section of the laser-welded NiTi was prepared using an EDM and followed standard procedures, as mentioned in the ASTM E3-11 (2017) standard [25]. The WM sample was mirror-polished and etched with a HNO_3_ + H_2_O + HF solution in the ratio of 4:5:1. X-ray diffraction (XRD) was used to determine the phases in the NiTi WM. A Pan Analytical Empyrean diffractometer (Malvern Panalytical GmbH, Kassel, Germany) measured the 2θ range of 20–100 degrees. It was equipped with CuKα radiation with a wavelength of 0.1546 nm. The XRD scanning was performed at 0.05 degrees per second with a step angle of 0.02 degrees. The hardness measurements were performed by following the ASTM E384-22 standard [26] using a Struers Duramin-4 Vickers Hardness Tester with an applied load of 500 g and a dwell time of 15 s. The position for the hardness measurement was chosen to be 200 mm below the top and bottom surfaces of the weld bead, with 500 µm and 100 µm, respectively, between subsequent indentations in the x and y axes. According to the ASTM E8/E8M-22 standard [27], tensile specimens of the NiTi BM and WM were prepared and tested using an 8801 servo hydraulic universal tensile testing machine (Make: Instron, Norwood, MA, USA) with a cross head speed of 1 mm/min. Three specimens from the NiTi BM and WM were tested to evaluate the average tensile properties. Potentiodynamic polarization testing in SBF at 37.5 °C, following the ASTM G61-86 (2018) standard [28], was used to investigate the localized corrosion performance of the NiTi BM and WM. Tafel plots were obtained using a classical three-electrode system (Make: ACM Hill AC Instruments, Grange-Over-Sands, UK). The composition and pH of the SBF are shown in Table 3. The corrosion experiments were performed three times to ascertain the average corrosion properties. Equation (1) was utilized to compute the corrosion rate [29].

Corrosion rate (CR) = 39.4 × (3.27 × 10^−3^ I_corr_ Ew/ρ) (mpy) (1)

where I_corr_ is the corrosion current density in mA/cm^2^, E_w_ is the material’s equivalent weight in g, and ρ is the material’s density in g/cm^3^.

The EIS measurements were also performed with a 10 mV RMS sinusoidal perturbation with reference to OCP within the frequency limit of 10^5^ Hz to 10^2^ Hz. Using the Zview software, version 3.2, EIS spectrums from the experiments were matched. The corrosion pits were analyzed using SEM and EDS techniques to analyze the morphology and distribution of various elements around the pits.

## 3. Results and Discussion

**Figure 1:**
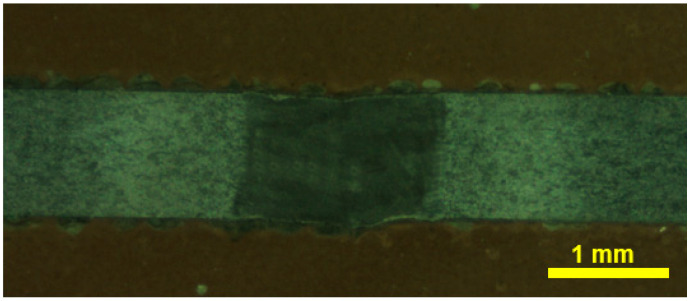
Macrograph of the laser-welded NiTi joint at 20× magnification.

**Figure 2:**
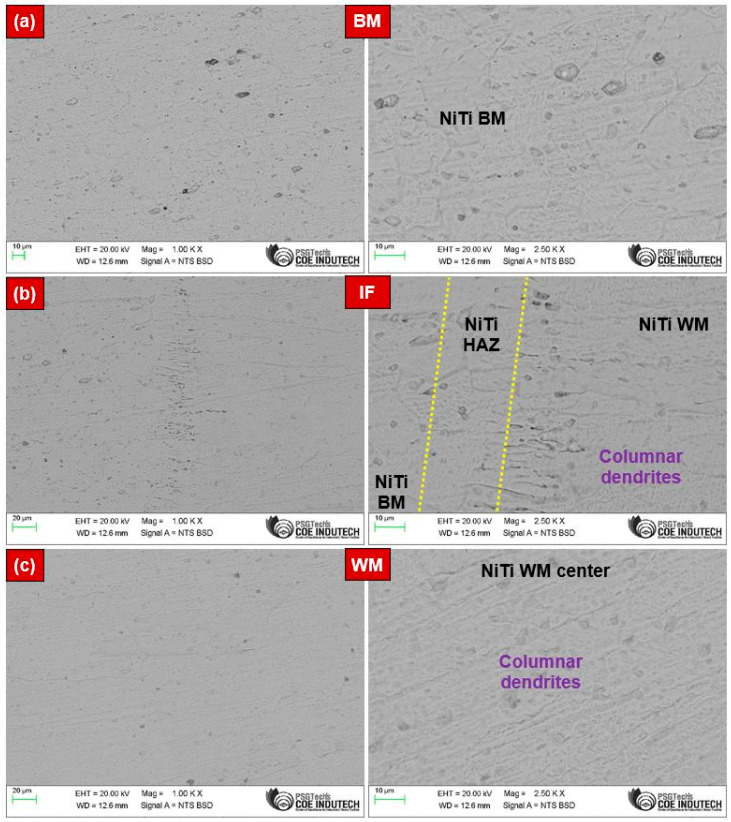
SEM micrographs of the laser-welded joint of NiTi at various locations: (**a**) BM, (**b**) HAZ, and (**c**) WM.

The macrostructure of the joint fabricated with optimized process parameters is shown in Figure 1. The laser-welded joint was devoid of defects such as cracks and had a uniform shape with full penetration. Figure 2 represents the SEM images of the laser-welded NiTi joint at different locations, specifically the BM, heat-affected zone (HAZ), and WM. Because of the higher temperature gradient experienced during laser-welding and the impact of solidification, the fusion zone (FZ) usually had a coarser grain structure than the HAZ. Due to the temperature difference at the center of FZ, the grains expanded, and the limited heat conductivity of NiTi alloys made it difficult for the heat to spread out relatively toward the candidate material.

Figure 2a shows the microstructure of the NiTi BM, dominantly austenitic with the B2 austenite phase. The FZ experienced epitaxial growth, with larger columnar dendrites aligned nearly perpendicular to the weld center line, as shown in Figure 2b. In contrast, coarse dendrites were noticed, as shown in Figure 2c. Figure 2b illustrates the interface region, highlighting the narrow HAZ within the dashed yellow lines because of a lower heat input during laser-welding. The grains in the HAZ underwent coarsening due to an insufficient heat input, as noticed in Figure 2b. Figure 2c presents the microstructure of the NiTi WM, mainly with columnar dendrites. The WM retained the B2 austenitic structure and formed Ti_2_Ni and Ni_4_Ti_3_ precipitates [30]. Identical microstructural characteristics were reported in earlier studies [6,31].

**Figure 3:**
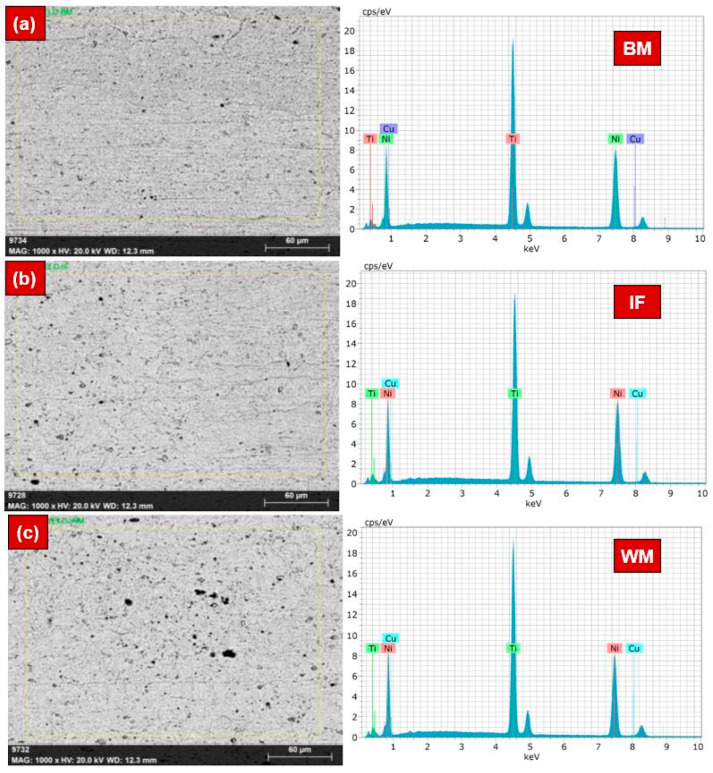
SEM images and EDS area scan plots at different locations in NiTi weldment: (**a**) BM, (**b**) interface, and (**c**) WM.

Considering the microstructural differences across the laser-welded NiTi, an EDS area scan analysis was performed at the BM, interface (IF), and WM regions, as shown in Figure 3. The IF region was comprised of the BM, HAZ, and WM (refer to Figure 3b). The concentrations of Ni, Ti, and Cu elements were obtained at different locations (refer to Table 4). There was not much difference in the concentration of Ni and Ti across the weldment. A minor difference was noticed, confirming the quality of laser-welded NiTi sheets and the formation of precipitates. The obtained EDS results align with Mehrpouya et al.’s previous observation [6]. As a result of the vaporization phenomenon during laser-welding, the wt% of Ni in the HAZ and WM marginally decreased [32].

Table 4 also makes it clear that there was very little variation in the weight percentage of Ni and Ti throughout the weld, i.e., lower heat input will have less influence on the weld, resulting in the preservation of functional properties. The difference in the Cu levels at different regions was attributed to the differences in the melting point, solubility, and partition coefficients compared to other elements. During welding, the rapid solidification may have been distributed differently in various regions of the NiTi WM.

**Figure 4:**
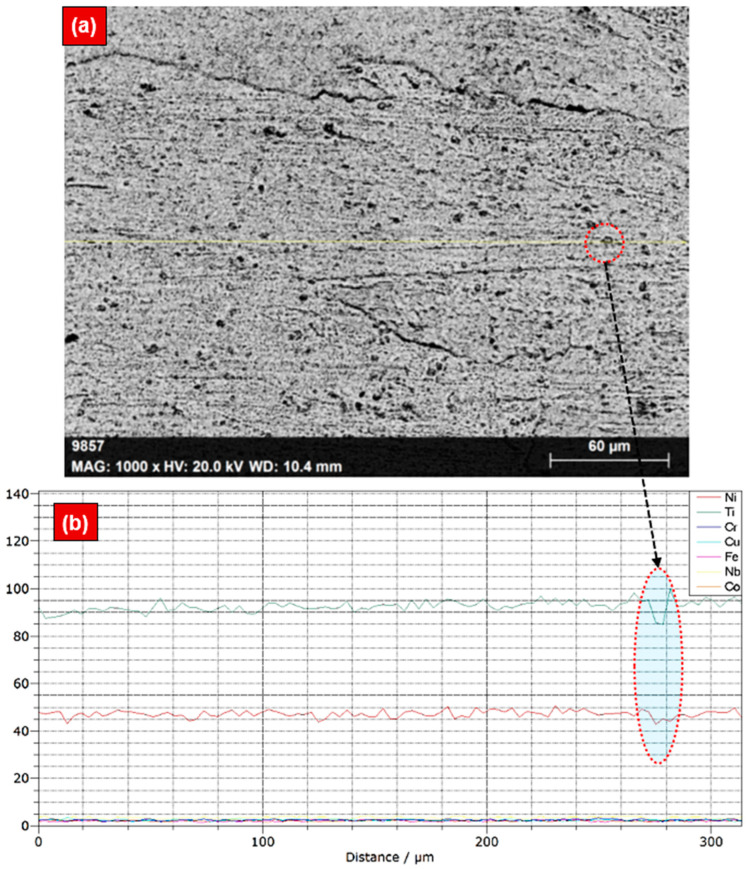
SEM-EDS responses of the NiTi WM: (**a**) SEM image and (**b**) EDS line scan plots.

Figure 4 shows the SEM image and EDS line scan at the WM, highlighting the concentration of Ni and Ti elements. The area highlighted by the red dotted lines indicates that metastable Ti_2_Ni and Ni_4_Ti_3_ precipitated phases were present in the WM. These phases have been previously reported in earlier studies [33]. Hence, the decline in Ni and increase in Ti was noticed within the WM region compared to the BM, while the same can be confirmed from the EDS line-mapping spectra. The increase in the Ti was attributed to the recirculation of the molten pool during laser-welding [24].

**Figure 5:**
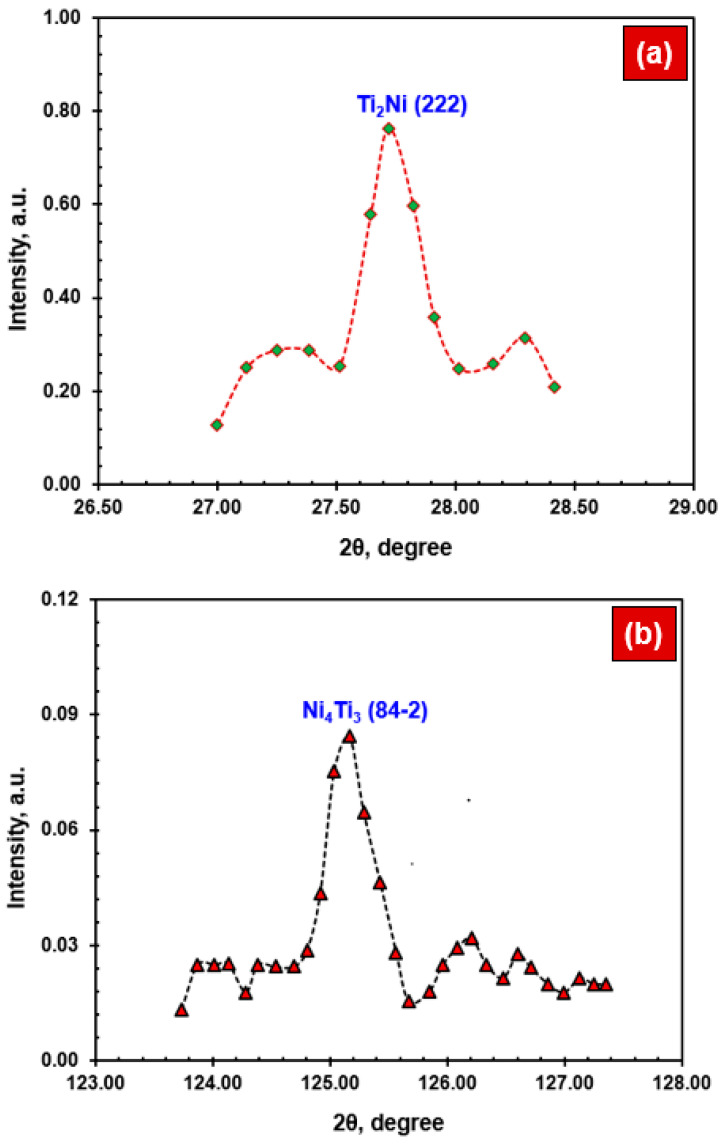
XRD analysis of NiTi WM confirming the intermetallic phases: (**a**) Ti_2_Ni and (**b**) Ni_4_Ti_3_.

Figure 5 shows the XRD spectra of different intermetallic phases formed in the NiTi specimen after laser-welding. Equiatomic NiTi was the main phase, along with the intermetallic precipitates, such as Ti_2_Ni (refer to Figure 5a) and Ni_4_Ti_3_ (refer to Figure 5b). The presence of Ti_2_Ni affected the mechanical properties. In contrast, the existence of Ni_4_Ti_3_ affected the phase transformation temperatures and mechanical characteristics. The NiTi WM samples clearly showed two distinct peaks of intermetallic precipitates, including Ti_2_Ni (222) and Ni_4_Ti_3_ (84-2). This observation was similar to the results obtained in the previous study [24,34].

**Figure 6:**
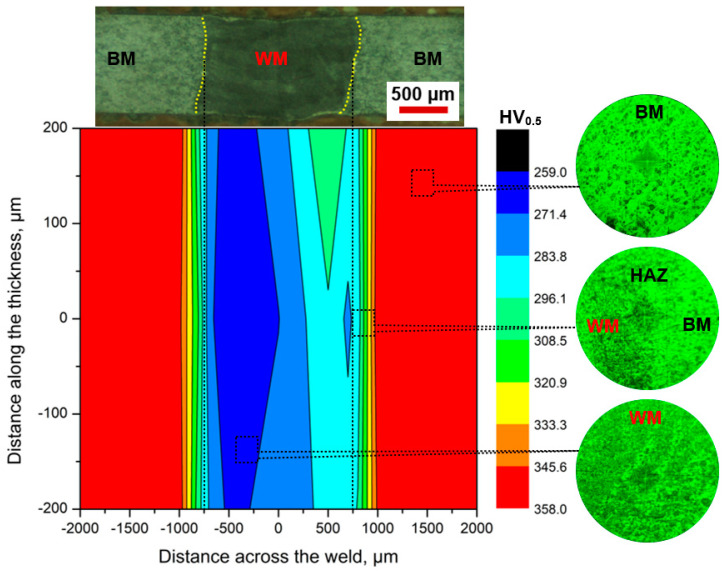
Contour plot showing the hardness variation across the laser-welded NiTi joint.

The hardness variation across the NiTi weldment is shown by the contour plot along with the microstructure and indentations at different zones in inset images (refer to Figure 6). The macrograph was matched with the contour map to highlight the hardness variation at the BM, HAZ, and WM. The average hardness in the NiTi BM was 352 ± 5 HV. Because of the microstructural variations throughout the NiTi weldment, the microhardness value progressively decreased from the BM to the WM center through HAZ. The hardness values in the HAZ and WM ranged between 275 and 307 HV and 265 and 287 HV, respectively. The grain coarsening in HAZ and dendrites in the WM reduced the hardness considerably. During solidification, the nucleation and growth of dendrites, the formation of Ti_2_Ni and Ni_4_Ti_3_ precipitates, and the recrystallization of HAZ influenced the hardness in the NiTi joint. Identical trends in hardness measurements have been reported in earlier studies [16,35].

**Figure 7:**
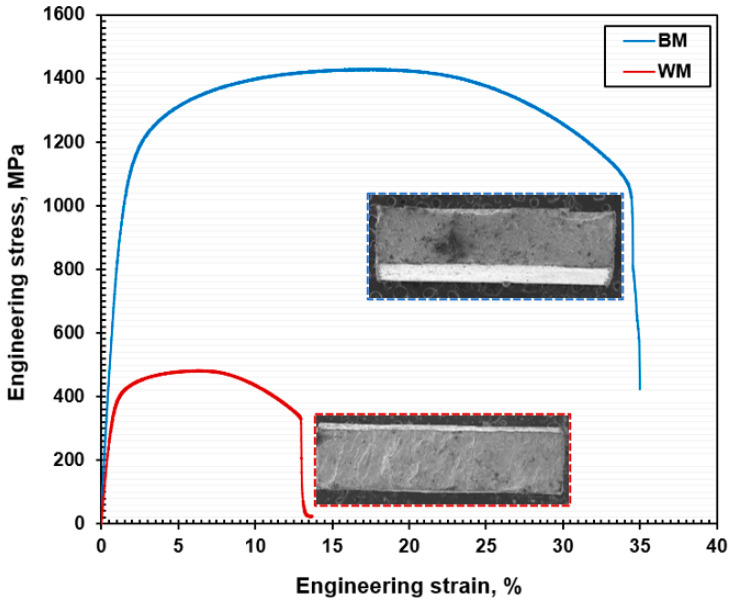
Engineering stress vs stress curves of the NiTi BM and WM specimens.

Figure 7 represents the engineering stress vs strain curves of BM and WM specimens of NiTi. The average tensile characteristics reported in Table 5 were determined by testing three samples each from the BM and WM. The graphs show that the WM specimens’ tensile strength (UTS) and percentage of elongation (EL) were much lower than those of the BM specimens. The average UTS and EL of the BM specimen was 1430 ± 34 MPa and 34.50 ± 1%, respectively. In contrast, the WM samples had a UTS and EL of 481 ± 19 MPa and 13.60 ± 0.5%, correspondingly due to the microstructural difference. The microstructural difference and the formation of precipitated phases with Ni and Ti after laser-welding corroborate the considerable decrease in the tensile properties. The BM and WM NiTi specimens ruptured at the sample’s center. Identical trends have been reported in the previous studies focused on NiTi alloys via laser-welding [16].

**Figure 8:**
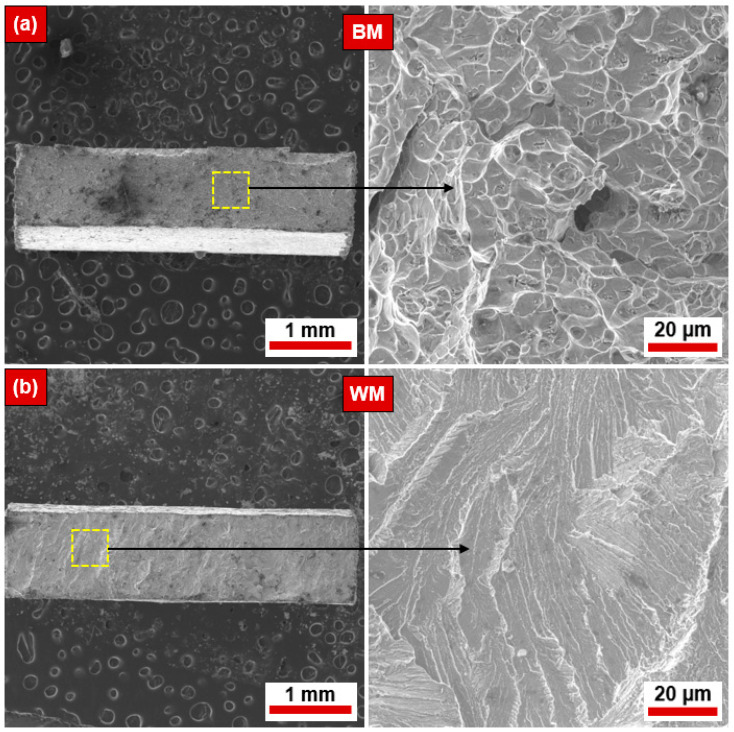
The fracture surface of the ruptured tensile specimens of NiTi: (**a**) BM and (**b**) WM.

The SEM micrographs of the fractured tensile specimens of the NiTi BM and WM are illustrated in Figure 8a and Figure 8b, respectively. The fracture mechanism in the BM is ductile in nature with dimples and micro-voids, as the ductility is better. However, the WM specimen underwent a drastic reduction in ductility, and the same can be confirmed by the ruptured surface characteristics (refer to Figure 8b). The fracture mechanism in the WM was brittle in nature with transgranular cleavage facets. No significant flaws were found on the BM and WM ruptured surfaces.

**Figure 9:**
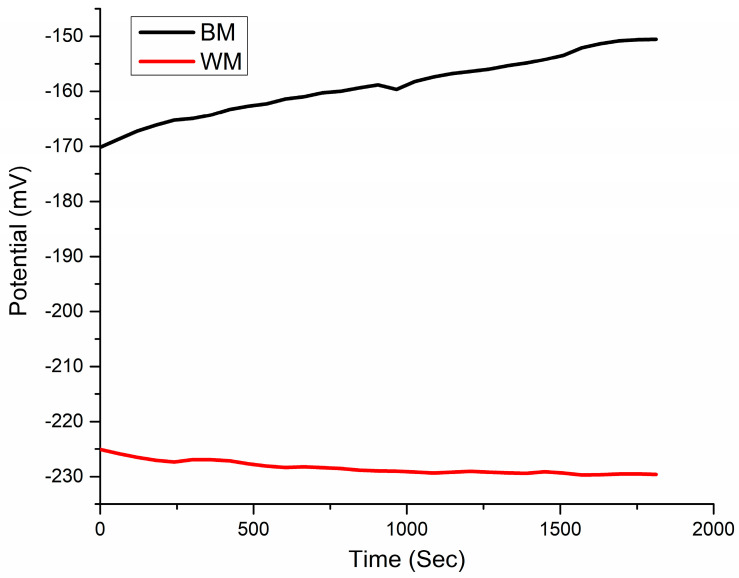
Variation between the BM and WM’s OCP overtime in SBF.

Electrochemical corrosion tests have been used to evaluate the localized corrosion resistance of the NiTi BM and WM using corrosion potential measurements. The NiTi BM and WM specimens’ open circuit potential (OCP) in an SBF environment is plotted against time in Figure 9. Approximately 55 mV was the OCP difference between the NiTi BM and the NiTi WM. This suggests that the formation of a protective oxide layer on the NiTi BM sample was more stable than that of the oxide layer that developed on the WM. In addition, there were no spikes in the plot, highlighting the absence of metastable pits.

**Figure 10:**
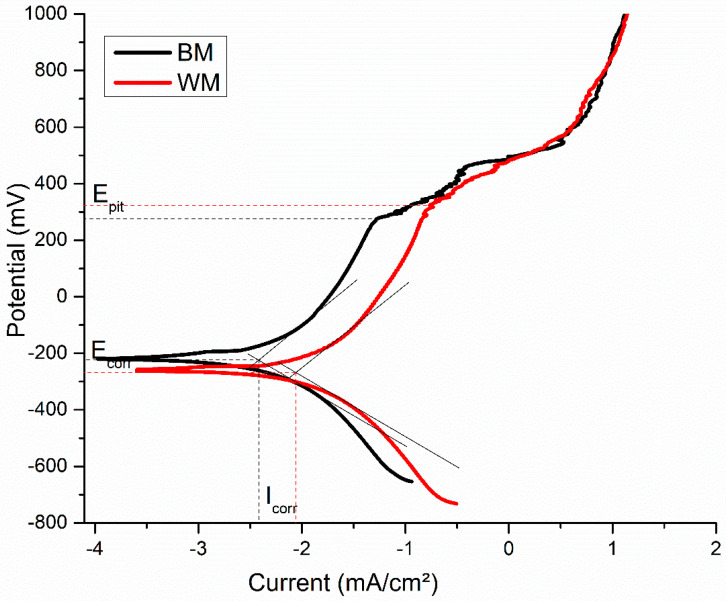
Potentiodynamic polarization curves of the NiTi BM and WM specimens.

Figure 10 shows the potentiodynamic polarization (PDP) curves, and Table 6 describes the corrosion potentials of NiTi BM and WM samples in SBF. It indicates the localized passivation behavior of the NiTi BM and WM specimens from the corrosion current density (I_corr_) measurements. The sample that exhibited a higher corrosion potential (E_corr_) and lower Icorr exhibited superior passivation performance in corrosive environments [36]. The NiTi BM sample had better corrosion resistance than the WM sample. For both the BM and WM specimens of NiTi, the Tafel plot shifted from the cathodic to the anodic regions, with different E_corr_ and I_corr_ values. Evidently, laser-welding reduced the resistance to corrosion in the NiTi alloys, as indicated by a lower E_corr_, higher I_corr_, lower E_pit_, and higher corrosion rate, as reported in Table 4. The pitting potential (E_pit_) of the NiTi BM and WM were 281.43 mV and 330.05 mV, respectively. According to the corrosion principle, the specimen with a positive E_pit_ value has a high level of resistance to localized corrosion and can withstand the breakdown of passive film [37]. The lower E_pit_ value of NiTi BM highlights the superior resistance to localized corrosion compared to the WM. The average corrosion rate of the NiTi BM and WM specimens in mils penetration per year (mpy) was 0.048 ± 0.0018 mpy and 0.41 ± 0.019 mpy, respectively. The corrosion rate was higher in the WM than in the BM specimen, and it mainly progressed in the Ti-enriched region. Fontana’s classifications indicate that a corrosion rate of less than 1 mpy is outstanding for the most widely used stainless steels and nickel-based superalloys, hence confirming the superior corrosion performance of the NiTi BM and WM [38].

**Figure 11:**
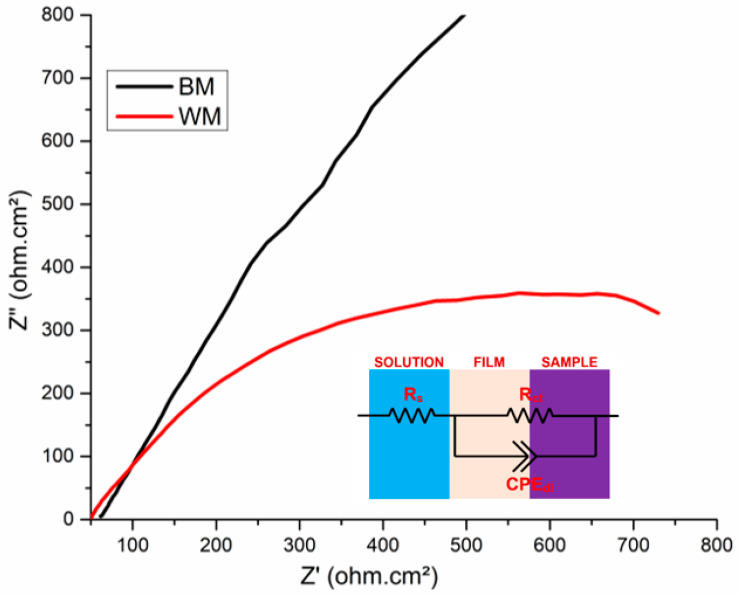
Nyquist plots of BM and laser-welded NiTi in SBF.

Using Nyquist plots (refer to Figure 11) obtained from the electrochemical impedance spectroscopy (EIS) analysis, the electrochemical behaviors of the NiTi BM and WM were examined. The diameter of the semicircles of the NiTi BM sample was significantly bigger than that of the WM, as can be seen from the Nyquist spectra displayed in Figure 11. The inset graphic in Figure 11 illustrates the equivalent circuit utilized for fitting the Nyquist curves. This indicates that NiTi BM had superior corrosion resistance to the NiTi WM sample. The capacitive spectra of NiTi WM confirm the lower corrosion resistance as a result of welding. The Nyquist spectra’s semi-circular capacitive arc indicates the passive film formation on the specimen surface, confirming the capacitance characteristics [29,39]. The corroded electrode’s maximum charge transfer resistance is highlighted by the larger capacitance radius, which also exhibited superior corrosion resistance [40]. The Nyquist spectra, OCP, and PDP curves of the NiTi specimens showed a good correlation.

**Figure 12:**
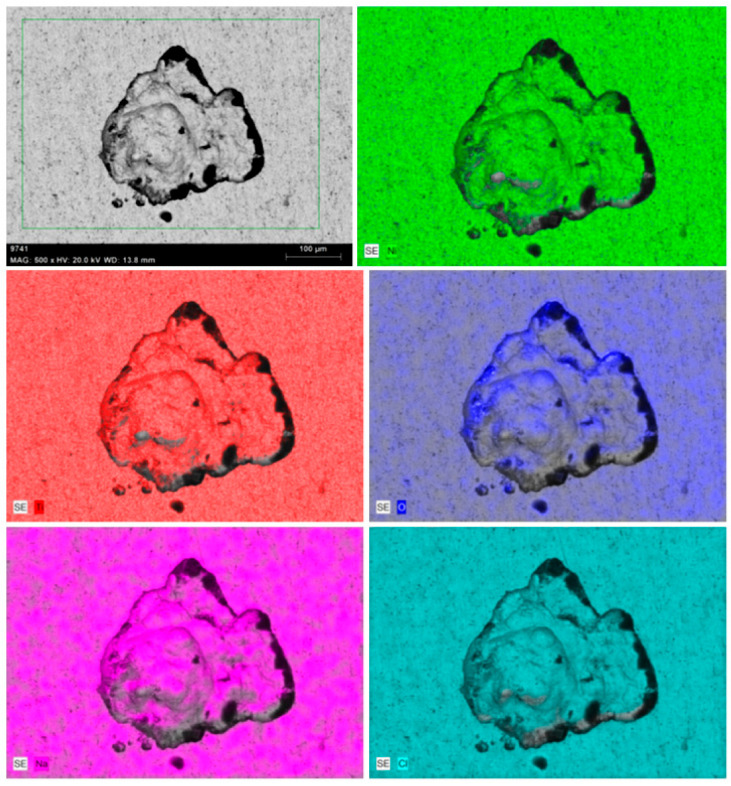
SEM image and EDS elemental maps of NiTi BM specimen after corrosion in SBF.

**Figure 13:**
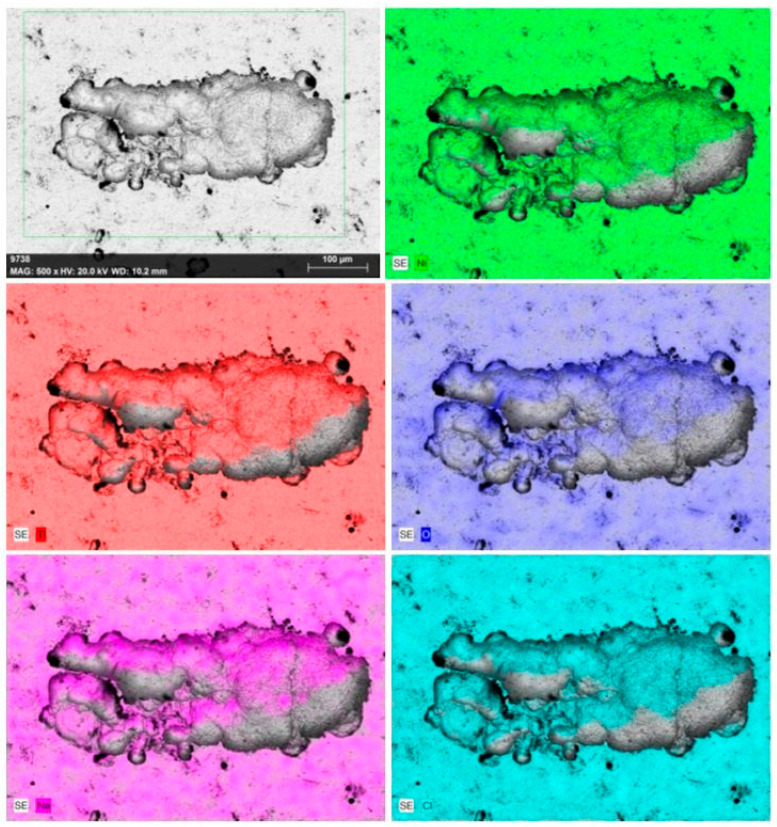
SEM image and EDS elemental maps of NiTi WM specimen after corrosion in SBF.

Figure 12 and Figure 13 show the SEM images of the pits and their corresponding EDS elemental maps around the pits after corrosion tests. Pits were noticed in both the NiTi BM and WM specimens. The size of the pits was less in the BM compared to the WM sample, and this is in good agreement with the corrosion rate. EDS elemental maps confirmed the depletion of Ni and Ti from the specimen surface exposed to an SBF environment, and the formation of oxides can be confirmed. Evidently, the release of Ni was confirmed when the metals were exposed to SBF environments for a prolonged duration. The formation of TiO_2_ oxides in the BM provides higher corrosion resistance, while the formation of precipitates, along with a coarse microstructure, affects the corrosion resistance of the WM. The main elements present in the corroded surface were nickel (Ni), titanium (Ti), oxygen (O), sodium (Na), and chlorine (Cl). The elements other than Ni and Ti on the specimen surface were corroborated by the interaction between the SBF and NiTi samples, resulting in the formation of oxides. The presence of O was noticed in the Ni- and Ti-depleted regions around the pits. A severely corroded region could be observed in the NiTi WM, as shown in Figure 12. From a previous study, it was noticed that the passive film formed on the NiTi BM and WM specimens was mainly comprised of highly stable TiO_2_ and acted as a barrier against the oxidation of Ni [41].

## 4. Conclusions

NiTi alloys are often limited in the manufacturing of small and miniature biomedical devices as a result of their poor formability and machinability characteristics. Laser-welding is one of the best techniques for fabricating defect-free NiTi components. The microstructural features, mechanical integrity, and localized electrochemical corrosion behavior of NiTi shape memory alloys in SBFs were evaluated, and the following inferences were drawn:

- The NiTi shape memory alloy sheets were successfully welded using laser-welding. A quality weld bead was obtained with a full penetration depth using the following process variables: laser power = 950 W; focal position = +0.50 mm; welding speed = 200 mm/min; shielding gas flow rate = 15 L/min.
- The NiTi BM mainly comprised B2 austenite phases within the austenitic matrix, while the NiTi WM showed the existence of B2 austenite phases along with coarse dendrites and precipitates such as Ti_2_Ni and Ni_4_Ti_3_. A narrow HAZ was noticed, i.e., <50 µm.
- EDS area mapping confirmed the minor difference in the wt% of Ni and Ti in the WM, while the fraction of Ni and Ti in the BM and HAZ was almost identical. EDS line-mapping highlighted the existence of intermetallic precipitates within the WM region.
- The microhardness measurements showed an increasing trend from the WM center to the BM. The average hardness in the BM was 352 ± 5 HV, while the hardness in the HAZ and WM ranged between 275 and 307 HV and 265 and 287 HV, respectively.
- Uni-axial tensile tests revealed the reduction in tensile properties of the NiTi WM sample compared to the NiTi BM. The joint efficiency was approximately 34%, and this reduction in tensile strength is corroborated by the coarse dendrites in the FZ and the precipitation of intermetallic phases.
- The mode of ruptures in the NiTi BM was ductile, with the coalescence of dimples and microvoids. At the same time, the NiTi WM exhibited a brittle mode of rupture mechanisms with transgranular cleavage facets.
- The OCP value of the NiTi BM specimen was 55 mV higher than the WM sample. The corrosion parameters derived from Tafel plots demonstrate that welding decreased the NiTi alloy’s resistance to corrosion in SBF. The corrosion rate in WM (0.41 ± 0.019 mpy) was higher than that of the NiTi BM (0.048 ± 0.0018 mpy).
- The Nyquist spectra illustrated the capacitance characteristics of the NiTi BM and WM, highlighting the corrosion performance in SBF. This drastic reduction in the localized passive film formation in the WM was attributed to the development of an inhomogeneous oxide layer as a result of the weld’s coarse microstructure.
- SEM images confirmed the existence of pits in both the BM and WM samples of the NiTi alloy and EDS elemental mapping around the pit region highlighted the formation of oxides and the depletion of Ni and Ti. The pit size was considerably higher in the WM than in the BM specimen.

This experimental study demonstrates the potential use of welded NiTi parts in biomedical applications. The choice of suitable laser process variables controls the laser-welded samples’ overall mechanical and corrosion performance. The alteration in the microstructural features and formation of intermetallic precipitates affects the corrosion performance of NiTi specimens in biological environments. In addition, post-weld treatment or surface treatment shall be considered to improve welded NiTi components’ mechanical and electrochemical performance.

## Figures and Tables

**Table 1:** Chemical composition of NiTi sheets at.%.

Element	Ni	Cr	Fe	C	Nb	Co	O	Cu	N	H	Ti
at·%	55.12	0.005	0.012	0.038	0.015	0.05	0.035	0.005	0.001	0.001	Bal.

**Table 2:** The optimized combination of process variables for welding NiTi sheets.

Variable	Laser Power	Focal Position	Welding Speed	Shielding Gas
Unit	W	mm	mm/min	L/min
Value	950	+0.50	200	15

**Table 3:** Chemical composition and pH of the SBF solution.

Reagents	Amount in g/1000 mL	pH
NaCl	8.036	~7.4
NaHCO_3_	0.352	
KCl	0.225	
K_2_HPO_4_·3H_2_O	0.230	
MgCl_2_·6H_2_O	0.311	
1.0 M HCl	40 mL	
CaCl_2_·2H_2_O	0.293	
Na_2_SO_4_	0.072	
(HOCH_2_)3CNH_2_	6.063	
1.0 M HCl	An appropriate amount was added to adjust the pH	

**Table 4:** EDS point scan responses reveal the elemental composition in different regions.

Location	Elemental Composition in wt%		
	Ni	Ti	Cu
BM	55.12	44.23	0.006
IF	53.80	45.69	0.05
WM	53.45	46.12	0.03

**Table 5:** Tensile properties of wrought and welded specimens of NiTi.

Specimen Type	Tensile Strength, UTS	Percent of Elongation, EL	Fracture Location
Unit	MPa	%	
BM	1430 ± 34	34.50 ± 1	center
WM	481 ± 19	13.60 ± 0.5	center

**Table 6:** Results of PDP measurements for corrosion of BM and WM specimens in SBF.

Specimen Type	E_corr_	I_corr_	E_pit_	Corrosion Rate
Unit	mV	(mA/cm^2^)	mV	mpy
BM	−217.05 ± 4	−2.39 ± 0.10	281.43 ± 11	0.048 ± 0.0018
WM	−259.53 ± 9	−2.01 ± 0.09	330.05 ± 7	0.41 ± 0.019

## Data Availability

The original contributions presented in the study are included in the article; further data are available with the permission of all authors.
